# The law of the iterated logarithm for LNQD sequences

**DOI:** 10.1186/s13660-017-1607-5

**Published:** 2018-01-08

**Authors:** Yong Zhang

**Affiliations:** College of Mathematics, Jilin University, Changchun, 130012 P.R. China

**Keywords:** law of the iterated logarithm, linear process, Stein’s method, LNQD sequence, Beveridge and Nelson decomposition

## Abstract

Let $\{\xi_{i},i\in{\mathbb{Z}}\}$ be a stationary LNQD sequence of random variables with zero means and finite variance. In this paper, by the Kolmogorov type maximal inequality and Stein’s method, we establish the result of the law of the iterated logarithm for LNQD sequence with less restriction of moment conditions. We also prove the law of the iterated logarithm for a linear process generated by an LNQD sequence with the coefficients satisfying $\sum_{i=-\infty}^{\infty}|a_{i}|<\infty$ by a Beveridge and Nelson decomposition.

## Introduction

Two random variables *X* and *Y* are said to be negatively quadrant dependent (NQD, for short), if $P(X\leq x,Y\leq y)-P(X\leq x)P(Y\leq y)\leq0$ for all $x,y\in {\mathbb{R}}$. A sequence $\{X_{k},k\in{\mathbb{Z}}\}$ is said to be linear negatively quadrant dependent (LNQD, for short) if for any disjoint finite subsets $A,B\subset{\mathbb{Z}}$ and any positive real numbers $r_{j}$, $\sum_{i\in A}r_{i}X_{i}$ and $\sum_{j\in B}r_{j}X_{j}$ are NQD. It is obvious that LNQD implies NQD. The definitions of NQD and LNQD can be found in Lehmann [1] and Newman [2].

A much stronger concept than LNQD was introduced by Joag-Dev and Proschan [3]: for a finite index set *I*, the r.v.s. $\{X_{i},i\in I\}$ are said to be negatively associated (NA, for short), if for any disjoint nonempty subsets *A* and *B* of *I*, and any coordinatewise nondecreasing function *G* and *H* with $G: {\mathbb{R}}^{A}\rightarrow{\mathbb{R}}$ and $H: {\mathbb {R}}^{B}\rightarrow{\mathbb{R}}$ and $EG^{2}(X_{i},i\in A)<\infty$, $EH^{2}(X_{j},j\in B)<\infty$, we have $\operatorname{Cov} (G(X_{i},i\in A),H(X_{j},j\in B))\leq0$. An infinite family is NA if every finite subfamily is NA.

Some applications for LNQD sequence have been found. For example, Newman [2] established the central limit theorem for a strictly stationary LNQD process, Dong and Yang [4] provided the almost sure central limit theorem for an LNQD sequence, Wang and Zhang [5] provided uniform rates of convergence in the central limit theorem for LNQD sequence, Li and Wang [6] obtained the asymptotic distribution for products sums of LNQD sequence, Ko *et al.* [7] studied the strong convergence for weighted sums of LNQD arrays, Ko *et al.* [8] obtained the Hoeffding-type inequality for LNQD sequence, Zhang *et al.* [9] established an almost sure central limit theorem for products sums of partial sums under LNQD sequence, Wang *et al.* [10] discussed the exponential inequalities and complete convergence for an LNQD sequence, Choi [11] obtained the Limsup results and a uniform LIL for partial sums of an LNQD sequence, Wang and Wu [12] obtained the strong laws of large numbers for arrays of rowwise NA and LNQD random variables, Wang and Wu [13] established the central limit theorem for stationary linear processes generated by LNQD sequence, Li *et al.* [14] established some inequalities for LNQD sequence, Shen *et al.* [15] proved the complete convergence for weighted sums of LNQD sequence, and so forth. It is easily seen that independent random variables and NA random variables are LNQD. Since LNQD random variables are much weaker than independent random variables and NA random variables, studying the limit theorems for LNQD sequence is of interest.

The main purpose of this paper is to discuss the limit theory for LNQD sequence. In Section 2, by the Kolmogorov type maximal inequalities and Stein’s method, we obtain the law of the iterated logarithm for strictly stationary LNQD sequence with finite variance. In Section 3, we prove the law of the iterated logarithm for linear process generated by LNQD sequence with less restrictions by Beveridge and Nelson decomposition for linear process.

Throughout the paper, *C* denotes a positive constant, which may take different values whenever it appears in different expressions. We have $\log x=\ln{\max\{e,x\}}$.

## Main results

We will need the following property.

1. (Hoeffding equality): For any absolutely continuous functions *f* and *g* on ${\mathbb{R}}^{1}$ and for any random variables *X* and *Y* satisfying $Ef^{2}(X)+Eg^{2}(Y)<\infty$, we have
  $$\begin{aligned}[b] &\operatorname{Cov}\bigl(f(X),g(Y)\bigr)\\ &\quad= \int_{-\infty}^{\infty} \int_{-\infty }^{\infty}f'(x)g'(y) \bigl\{ P(X\geq x,Y\geq y)-P(X\geq x)P(Y\geq y)\bigr\} \,dx\,dy.\end{aligned} $$

Now we state the law of iterated logarithm for LNQD sequence.

### Theorem 2.1

*Let* $\{\xi_{i},i\geq1\}$ *be a strictly stationary LNQD sequence with* $E\xi_{i}=0$, $E\xi_{i}^{2}<\infty$ *and* $\sigma^{2}=E\xi_{1}^{2}+2\sum_{i=2}^{\infty}E\xi_{1}\xi_{i}>0$. *Put* $S_{n}=\sum_{i=1}^{n}\xi_{i}$. *Then*

2.1 $$ \limsup_{n\to\infty}\frac{S_{n}}{(2\sigma^{2}n\log\log n)^{1/2}}=1 \quad \textit{a.s.} $$

### Remark 2.2

Our theorem extends the corresponding results of Corollary 1.2 in Choi [11]. Choi established a law of the iterated logarithm for LNQD sequence with $E|\xi_{1}|^{2+\delta}<\infty$ for some $\delta>0$ and variance coefficients decaying polynomially. But our theorem only restricts the finite variance.

The proof of Theorem 2.1 is based on the following lemmas.

### Lemma 2.3

(Lehmann [1])

*Let random variables X and Y be NQD*, *then*

1. $EXY\leq EXEY$;
2. $P(X >x, Y> y) \leq P(X >x)P(Y> y)$;
3. *if* *f* *and* *g* *are both nondecreasing* (*or both nonincreasing*) *functions*, *then* $f(X)$ *and* $g(Y)$ *are NQD*.

### Lemma 2.4

*Let* $\{\xi_{i}, 1\leq1\leq n\}$ *be an LNQD sequence of random variables with mean zero and finite second moments*. *Let* $S_{n}=\sum_{i=1}^{n}\xi_{i}$ *and* $B_{n}=\sum_{i=1}^{n}E\xi_{i}^{2}$. *Then*, *for all* $x>0$, $a>0$ *and* $0<\alpha<1$, *we know*

2.2 $$\begin{aligned}& \begin{aligned}[b] &P\Bigl(\max_{1\leq k\leq n}S_{k}\geq x\Bigr) \\ &\quad\leq P\Bigl(\max_{1\leq k\leq n}\xi_{k}>a\Bigr) + \frac{1}{1-\alpha}\exp \biggl(-\frac{x^{2}\alpha}{2(ax+B_{n})} \biggl\{ 1+\frac {2}{3}\log \biggl(1+\frac{ax}{B_{n}}\biggr) \biggr\} \biggr) \\ &\quad\leq P\Bigl(\max_{1\leq k\leq n}\xi_{k}>a\Bigr)+ \frac{1}{1-\alpha}\exp \biggl(-\frac{x^{2}\alpha }{2(ax+B_{n})} \biggr),\end{aligned} \end{aligned}$$

2.3 $$\begin{aligned}& \begin{aligned}[b] &P\Bigl(\max_{1\leq k\leq n}|S_{k}| \geq x\Bigr) \\ &\quad\leq 2P\Bigl(\max_{1\leq k\leq n}|\xi_{k}|>a\Bigr) +\frac{2}{1-\alpha}\exp \biggl(-\frac{x^{2}\alpha}{2(ax+B_{n})} \biggl\{ 1+\frac {2}{3}\log \biggl(1+\frac{ax}{B_{n}}\biggr) \biggr\} \biggr) \\ &\quad\leq 2P\Bigl(\max_{1\leq k\leq n}|\xi_{k}|>a\Bigr)+ \frac{2}{1-\alpha}\exp \biggl(-\frac{x^{2}\alpha }{2(ax+B_{n})} \biggr).\end{aligned} \end{aligned}$$

*In particular*, *we have*

2.4 $$ \begin{aligned}[b] &P\Bigl(\max_{1\leq k\leq n}|S_{k}| \geq x\Bigr) \\ &\quad\leq 2P\Bigl(\max_{1\leq k\leq n}|\xi_{k}|>a\Bigr) +4\exp \biggl(-\frac{x^{2}}{8B_{n}} \biggr)+4 \biggl(\frac{B_{n}}{4(ax+B_{n})} \biggr)^{x/{(12a)}}.\end{aligned} $$

### Proof

By Lemma 2.3, following the proof of Theorem 3 in Shao [16], we can easily get the results of Lemma 2.4. □

### Lemma 2.5

*Let* $\{Y_{i}, 1\leq i\leq n\}$ *be an LNQD sequence of random variables with* $EY_{i}=0$ *and* $E|Y_{i}|^{3}<\infty$. *Define* $T_{n}=\sum_{i=1}^{n}Y_{i}$ *and* $B_{n}^{2}=\sum_{i=1}^{n}EY_{i}^{2}$. *Then*, *for any* $x>0$,

$$ P(T_{n}\geq xB_{n})\geq\bigl(1-\Phi(x+1) \bigr)+6B_{n}^{-2}\sum_{1\leq i\neq j\leq n}E(Y_{i}Y_{j})-12B_{n}^{-3} \sum_{i=1}^{n}E|Y_{i}|^{3}, $$

*where* Φ *is the standard normal distribution function*.

### Proof

We will apply the Stein method. Let *X* be a standard normal random variable and define

$$g(w)=\left \{ \textstyle\begin{array}{l@{\quad}l} 0,&\text{for } w< x,\\ w-x, & \text{for } x\leq w\leq x+1, \\ 1,&\text{for } w> x+1. \end{array}\displaystyle \right . $$

Let *f* be the unique bounded solution of the Stein equation

2.5 $$ f'(w)-w f(w)=g(w)-Eg(X). $$

The solution *f* is given by

$$ f(w)=e^{w^{2}/2} \int_{-\infty}^{w}\bigl\{ g(t)-Eg(X)\bigr\} e^{-t^{2}/2}\,dt. $$

It is well known that (see Stein [17])

2.6 $$ \big|f(w)\big|\leq2,\qquad \big|f'(w)\big|\leq2,\quad\quad \big|f''(w)\big| \leq2. $$

Let $\zeta_{i}=Y_{i}/{B_{n}}$, $W=\sum_{i=1}^{n}\zeta_{i}$, $W^{(i)}=W-\zeta_{i}$,

$$\begin{gathered} \zeta_{i,1}=\left \{ \textstyle\begin{array}{l@{\quad}l} -1,&\text{for } \zeta_{i}< -1,\\ \zeta_{i}, & \text{for } {-}1\leq\zeta_{i} \leq1, \\ 1,&\text{for } \zeta_{i}> 1, \end{array}\displaystyle \right . \\\zeta_{i,2}=\left \{ \textstyle\begin{array}{l@{\quad}l} \zeta_{i}+1,&\text{for } \zeta_{i}< -1,\\ 0, & \text{for } {-}1\leq\zeta_{i}\leq1, \\ \zeta_{i}-1,&\text{for } \zeta_{i}> 1. \end{array}\displaystyle \right .\end{gathered} $$

Obviously, $\zeta_{i}=\zeta_{i,1}+\zeta_{i,2}$. Write

2.7 $$ \begin{aligned}[b] E\bigl(Wf(W)\bigr)&=\sum_{i=1}^{n}E \bigl(\zeta_{i}f\bigl(W^{(i)}\bigr)\bigr)+\sum _{i=1}^{n}E\bigl\{ \zeta_{i}\bigl[f(W)-f \bigl(W^{(i)}\bigr)\bigr]\bigr\} \\ &=:R_{1}+R_{2}+R_{3}+R_{4},\end{aligned} $$

where

$$\begin{gathered} R_{1}=\sum_{i=1}^{n}E\bigl( \zeta_{i}f\bigl(W^{(i)}\bigr)\bigr),\qquad R_{2}=\sum _{i=1}^{n}E\bigl\{ \zeta _{i,2} \bigl[f(W)-f\bigl(W^{(i)}\bigr)\bigr]\bigr\} , \\R_{3}=\sum_{i=1}^{n}E\bigl\{ \zeta_{i,1}\bigl[f\bigl(W^{(i)}+\zeta_{i,1}+\zeta _{i,2}\bigr)-f\bigl(W^{(i)}+\zeta_{i,1}\bigr)\bigr] \bigr\} , \\R_{4}=\sum_{i=1}^{n}E\bigl\{ \zeta_{i,1}\bigl[f\bigl(W^{(i)}+\zeta_{i,1}\bigr)-f \bigl(W^{(i)}\bigr)\bigr]\bigr\} .\end{gathered} $$

By the definition of LNQD, we know $\zeta_{i}$ and $W^{(i)}$ are NQD, then by (H1) and (2.6) we have

$$ |R_{1}|\leq\sum_{i=1}^{n}\big| \operatorname{Cov}\bigl(\zeta_{i},f\bigl(W^{(i)}\bigr)\bigr)\big| \leq2\sum_{i=1}^{n}\big|\operatorname{Cov}\bigl( \zeta_{i},W^{(i)}\bigr)\big| =-2B_{n}^{-2}\sum _{1\leq i\neq j\leq n}E(Y_{i}Y_{j}). $$

By (2.6), we obtain

$$\begin{gathered} |R_{2}|\leq4\sum_{i=1}^{n} E| \zeta_{i,2}| \leq4\sum_{i=1}^{n} E| \zeta_{i}|^{3}=4B_{n}^{-3}\sum _{i=1}^{n} E|Y_{i}|^{3}, \\|R_{3}|\leq2\sum_{i=1}^{n} E| \zeta_{i,1}\zeta_{i,2}|\leq2\sum_{i=1}^{n} E|\zeta_{i,2}| \leq2B_{n}^{-3}\sum _{i=1}^{n} E|Y_{i}|^{3}.\end{gathered} $$

To estimate $R_{4}$, let $K_{i}(t)=E(\zeta_{i,1}I\{0\leq t\leq\zeta_{i,1}\} -\zeta_{i,1}I\{\zeta_{i,1}\leq t< 0\})$. Rewrite $R_{4}$ as

$$\begin{aligned} R_{4}={}&\sum_{i=1}^{n}E \biggl\{ \zeta_{i,1} \int_{0}^{\zeta _{i,1}}f'\bigl(W^{(i)}+t \bigr)\,dt \biggr\} \\ ={}&\sum_{i=1}^{n} \int_{-1}^{1}E\bigl\{ f' \bigl(W^{(i)}+t\bigr)\bigl[\zeta_{i,1}I\{0\leq t\leq \zeta_{i,1}\}-\zeta_{i,1}I\{\zeta_{i,1}\leq t< 0\} \bigr]\bigr\} \,dt \\ ={}&\sum_{i=1}^{n} \int_{-1}^{1}E\bigl\{ f' \bigl(W^{(i)}+t\bigr)\bigr\} K_{i}(t)\,dt+\sum _{i=1}^{n} \int _{0}^{1}\operatorname{Cov} \bigl(f'\bigl(W^{(i)}+t\bigr),\zeta_{i,1}I\{0\leq t\leq\zeta _{i,1}\}\bigr)\,dt \\ &-\sum_{i=1}^{n} \int_{-1}^{0}\operatorname{Cov}\bigl(f' \bigl(W^{(i)}+t\bigr),\zeta _{i,1}I\{\zeta_{i,1}\leq t< 0\}\bigr)\,dt \\ =:{}&R_{4,1}+R_{4,2}+R_{4,3}.\end{aligned} $$

For fixed $0< t<1$, $xI\{0\leq t\leq x\}$ is a nondecreasing functions of *x*, by the definition of LNQD and Lemma 2.3, $\zeta_{i,1}I\{ 0\leq t\leq\zeta_{i,1}\}$ and $W^{(i)}$ are NQD. Then by (H1) and (2.7),

$$\begin{aligned} |R_{4,2}|&\leq2\sum_{i=1}^{n} \int_{0}^{1}\big|\operatorname {Cov} \bigl(W^{(i)},\zeta_{i,1}I\{0\leq t\leq\zeta_{i,1}\} \bigr)\big|\,dt \\ &\leq 2\sum_{i=1}^{n} \int_{0}^{1}\big|\operatorname {Cov} \bigl(W^{(i)},\zeta_{i}\bigr)\big|\,dt =2 \sum _{i=1}^{n}\big|\operatorname{Cov}\bigl(W^{(i)}, \zeta_{i}\bigr)\big|=-2B_{n}^{-2}\sum _{1\leq i\neq j\leq n}E(Y_{i}Y_{j}).\end{aligned} $$

Similarly,

$$ |R_{4,3}|\leq-2B_{n}^{-2}\sum _{1\leq i\neq j\leq n}E(Y_{i}Y_{j}). $$

Let $R_{5}=|R_{1}|+|R_{2}|+|R_{3}|+|R_{4,2}|+|R_{4,3}|$. Observe that

$$\int_{-1}^{1}K_{i}(t)=E \zeta_{i,1}^{2} \quad\text{and} \quad \int _{-1}^{1}|t|K_{i}(t)= \frac{1}{2}E|\zeta_{i,1}|^{3}. $$

It follows from (2.6) that

$$\begin{aligned} P(T_{n}\geq xB_{n})-\bigl(1-\Phi(1+x)\bigr)\geq{}& Eg(W)-Eg(X) \\ ={}&E f'(W)-E W f(W)\\ ={}&Ef'(W)-R_{4,1}-R_{1}-R_{2}-R_{3}-R_{4,2}-R_{4,3} \\ \geq{}&{-}R_{5}+E f'(W) \Biggl(1-\sum _{i=1}^{n}E\zeta_{i,1}^{2}\Biggr)+ \sum_{i=1}^{n}E\zeta_{i,1}^{2}Ef'(W)-R_{4,1} \\ ={}&{-}R_{5}+E f'(W)\sum _{i=1}^{n}E\bigl(\zeta_{i}^{2}- \zeta_{i,1}^{2}\bigr) \\ &+\sum_{i=1}^{n}E \biggl\{ \int_{-1}^{1} \bigl\{ f' \bigl(W^{(i)}+\zeta _{i}\bigr)-f' \bigl(W^{(i)}+t\bigr) \bigr\} K_{i}(t)\,dt \biggr\} \\ \geq{}&{-}R_{5}-2\sum_{i=1}^{n}E \zeta_{i}^{2}I\bigl\{ |\zeta_{i}|>1\bigr\} \\ &-2\sum _{i=1}^{n}E \biggl\{ \int_{-1}^{1}\bigl(|\zeta_{i}|+t\bigr)K_{i}(t)\,dt \biggr\} \\ \geq{}&{-}R_{5}-2\sum_{i=1}^{n}E| \zeta_{i}|^{3}-4\sum_{i=1}^{n}E| \zeta _{i}|^{3} \\ ={}&{-}R_{5}-6B_{n}^{-3}\sum _{i=1}^{n} E|Y_{i}|^{3}. \end{aligned}$$

Finally, by putting the above inequalities together, we complete the proof of Lemma 2.5. □

### Proof of Theorem 2.1

It suffices to show that for $0<\varepsilon<\frac{1}{30}$

2.8 $$ \limsup_{n\to\infty}\frac{|S_{n}|}{(2\sigma^{2}n\log\log n)^{1/2}}\leq 1+8 \varepsilon \quad\text{a.s.} $$

and

2.9 $$ \limsup_{n\to\infty}\frac{|S_{n}|}{(2\sigma^{2}n\log\log n)^{1/2}}\geq 1-8 \varepsilon \quad\text{a.s.} $$

Let *m* be an integer such that

2.10 $$ \sigma_{m}^{2}=:E\xi_{1}^{2}+2 \sum_{i=2}^{m}E\xi_{1} \xi_{i}\leq\sigma^{2}(1+\varepsilon). $$

Put $a_{i}=\varepsilon\sigma(i/\log\log i)^{1/2}/m$. Define

$$\begin{gathered} g_{1}(a,x)=xI\bigl\{ |x|\leq a\bigr\} +a I\{x>a\}-a I\{x< -a\}, \\ g_{2}(a,x)=(x-a) I\{x>a\}+(x+a) I\{x< -a\}, \\ Y_{i,l}=g_{l}(a_{i},\xi_{i})-Eg_{l}(a_{i}, \xi_{i}),\qquad S_{i,l}=\sum_{j=1}^{i}Y_{j,l},\quad \text{for } l=1,2, \\ u_{i}=\sum_{j=(i-1)m+1}^{im}Y_{j,l} \quad\text{and}\quad U_{i}=\sum_{j=1}^{i}u_{j},\quad i=1,2,\ldots.\end{gathered} $$

It is obvious that $S_{n}=S_{n,1}+S_{n,2}$. By the same argument as of equation (2.2) from de Acosta [18], it is easy to check that

2.11 $$ \sum_{i=1}^{\infty}E| \xi_{i}|I\bigl\{ |\xi_{i}|>a_{i}\bigr\} /{(i\log\log i)^{1/2}}\leq C E\xi_{1}^{2}< \infty. $$

Hence, by Kronecker’s lemma

$$ \sum_{i=1}^{n}\frac{|\xi_{i}|I\{|\xi_{i}|>a_{i}\}+E|\xi_{i}|I\{|\xi_{i}|>a_{i}\} }{(n\log\log n)^{1/2}}\to0 \quad \text{a.s.} $$

and

2.12 $$ S_{n,2}/{(n\log\log n)^{1/2}}\to0 \quad \text{a.s.} $$

Observe that

2.13 $$ \begin{aligned}[b] \max_{1\leq i\leq n}|S_{i,1}|&\leq\max _{1\leq i\leq [n/m]}|U_{i}|+\max_{1\leq i\leq1+[n/m]}\sum _{j=(i-1)m+1}^{\min\{n,im\} }|Y_{j,1}| \\ &\leq \max_{1\leq i\leq[n/m]}|U_{i}|+ma_{n} \leq\max _{1\leq i\leq[n/m]}|U_{i}|+\varepsilon\sigma(n\log\log n)^{1/2}\end{aligned} $$

for every *n* sufficiently large,

$$ Eu_{i}^{2}/{\bigl(m\sigma_{m}^{2}\bigr)} \to1 \quad\text{as } i\to\infty $$

and

$$ \sum_{i=1}^{[n/m]}Eu_{i}^{2}/{ \bigl(n\sigma_{m}^{2}\bigr)}\to1 \quad\text{as } n\to\infty. $$

Hence, by (2.10)

2.14 $$ \sum_{i=1}^{[n/m]}Eu_{i}^{2} \leq\sigma^{2} (1+2\varepsilon)n $$

provided that *n* is sufficiently large.

By the definition of LNQD and Lemma 2.3, we know $\{u_{i},i\geq1\} $ are also LNQD random variables with $Eu_{i}=0$ and $|u_{i}|\leq2ma_{im}$ for every *i*. By Lemma 2.4 (with $\alpha =1-\varepsilon$, $a=2ma_{n}$), (2.13) and (2.14), we get

2.15 $$ \begin{aligned}[b] &P \Bigl( \max_{1\leq i\leq n}|S_{i,1}| \geq(1+8\varepsilon ) \bigl(2\sigma^{2}n\log\log n\bigr)^{1/2} \Bigr) \\ &\quad\leq P \Bigl( \max_{1\leq i\leq[n/m]}|U_{i}|\geq (1+7 \varepsilon) \bigl(2\sigma^{2}n\log\log n\bigr)^{1/2} \Bigr) \\ &\quad\leq\frac{2}{\varepsilon}\exp \biggl(-\frac{(1-\varepsilon )(1+7\varepsilon)^{2} \sigma^{2}n\log\log n}{(1+7\varepsilon)(2\sigma^{2}n\log\log n)^{1/2}2ma_{n}+\sum_{i=1}^{[n/m]}Eu_{i}^{2}} \biggr) \\ &\quad\leq\frac{2}{\varepsilon}\exp \biggl(-\frac{(1-\varepsilon )(1+7\varepsilon)^{2}\log\log n}{4(1+7\varepsilon)\varepsilon +1+2\varepsilon} \biggr) \\ &\quad\leq\frac{2}{\varepsilon}\exp \bigl(-(1+\varepsilon)\log\log n \bigr)\end{aligned} $$

for every sufficiently large *n*. By using the standard subsequence method, (2.15) yields

2.16 $$ \limsup_{n\to\infty}|S_{n,1}|/{\bigl(2 \sigma^{2}n\log\log n\bigr)^{1/2}}\leq 1+8\varepsilon \quad \text{a.s.} $$

Now (2.8) follows by (2.12) and (2.16).

To prove (2.9), let

$$ m_{k}=\bigl[2^{k^{1+\varepsilon}}\bigr],\qquad p_{k}= \bigl[k^{-2}2^{k^{1+\varepsilon}}\bigr],\qquad n_{k}=(m_{k}+p_{k})k^{4}. $$

It suffices to show that

2.17 $$ \sum_{k=1}^{\infty}P \bigl(S_{n_{k},1}\geq(1-7\varepsilon) \bigl(2\sigma^{2} n_{k}\log \log n_{k}\bigr)^{1/2}\bigr)=\infty. $$

In fact, by Lemma 2.4, similar to the proof of (2.15), we obtain

$$ \sum_{k=1}^{\infty}P\bigl(S_{n_{k-1},1}\geq \varepsilon\bigl(2\sigma^{2} n_{k}\log \log n_{k} \bigr)^{1/2}\bigr)< \infty. $$

Then by (2.17), we have

2.18 $$ \begin{aligned}[b] &\sum_{k=1}^{\infty}P \bigl(S_{n_{k},1}-S_{n_{k-1},1}\geq (1-8\varepsilon) \bigl(2 \sigma^{2} n_{k}\log\log n_{k} \bigr)^{1/2}\bigr) \\ &\quad\geq\sum_{k=1}^{\infty}P \bigl(S_{n_{k},1}\geq(1-7\varepsilon ) \bigl(2\sigma^{2} n_{k}\log\log n_{k}\bigr)^{1/2}\bigr) \\ &\qquad- \sum_{k=1}^{\infty}P\bigl(S_{n_{k-1},1} \geq\varepsilon\bigl(2\sigma^{2} n_{k}\log \log n_{k}\bigr)^{1/2}\bigr) \\ &\quad=\infty.\end{aligned} $$

By the definition of LNQD and Lemma 2.3, we see that $\{S_{n_{k},1}-S_{n_{k-1},1}, k\geq1\}$ is an LNQD sequence, then, for any $x>0$, $y>0$, $k\neq j$,

$$\begin{aligned} P(S_{n_{k},1}-S_{n_{k-1},1}\geq x, S_{n_{j},1}-S_{n_{j-1},1} \geq y) \leq P(S_{n_{k},1}-S_{n_{k-1},1}\geq x)P(S_{n_{j},1}-S_{n_{j-1},1} \geq y).\end{aligned} $$

Hence, by the generalized Borel-Cantelli lemma (see, *e.g.*, Kochen and Stone [19]), (2.18) yields

$$ \limsup_{k\to\infty}\frac{S_{n_{k},1}-S_{n_{k-1},1}}{(2\sigma^{2} n_{k}\log \log n_{k})^{1/2}}\geq1-8\varepsilon \quad \text{a.s.}, $$

which together with (2.8) and (2.12) gives

$$ \limsup_{k\to\infty}\frac{S_{n_{k}}}{(2\sigma^{2} n_{k}\log\log n_{k})^{1/2}}\geq1-8\varepsilon \quad \text{a.s.} $$

and hence (2.9) holds.

To verify (2.17), set

$$\begin{gathered} v_{i,1}=\sum_{j=(i-1)(m_{k}+p_{k})+1}^{(i-1)(m_{k}+p_{k})+m_{k}}Y_{j,1},\qquad v_{i,2}=\sum_{j=(i-1)(m_{k}+p_{k})+m_{k}+1}^{i(m_{k}+p_{k})}Y_{j,1},\quad 1\leq i\leq k^{4}, \\ T_{k,1}=\sum_{i=1}^{k^{4}}v_{i,1},\qquad T_{k,2}=\sum_{i=1}^{k^{4}}v_{i,2}.\end{gathered} $$

Obviously, $S_{n_{k},1}=T_{k,1}+T_{k,2}$. Then by Lemma 2.4, similar to the proof of (2.15), we obtain

$$ \sum_{k=1}^{\infty}P\bigl(T_{k,2}\geq \varepsilon\bigl(2\sigma^{2} n_{k}\log\log n_{k} \bigr)^{1/2}\bigr)< \infty. $$

Thus, we only need to show that

2.19 $$ \sum_{k=1}^{\infty}P \bigl(T_{k,1}\geq(1-6\varepsilon) \bigl(2\sigma^{2} n_{k}\log\log n_{k}\bigr)^{1/2}\bigr)=\infty. $$

It is easy to see that

$$ \frac{B_{k^{4}}^{2}}{n_{k}\sigma^{2}}=\sum_{i=1}^{k^{4}} \frac {Ev_{i,1}^{2}}{n_{k}\sigma^{2}}\to1 \quad\text{as } k\to\infty. $$

From Lemma 2.5, we obtain

2.20 $$ \begin{aligned}[b] &P\bigl(T_{k,1}\geq(1-6\varepsilon) \bigl(2 \sigma^{2} n_{k}\log\log n_{k} \bigr)^{1/2}\bigr) \\ &\quad\geq \bigl(1-\Phi\bigl(1+(1-5\varepsilon) (2\log\log n_{k})^{1/2} \bigr)\bigr) -J_{k,1}-J_{k,2},\end{aligned} $$

where

$$ J_{k,1}=6B_{k^{4}}^{-2}\sum _{1\leq i\neq j\leq k^{4}}|Ev_{i,1}v_{j,1}|,\qquad J_{k,2}=12B_{k^{4}}^{-3/2} \sum_{i=1}^{k^{4}}E|v_{i,1}|^{3},\quad B_{k^{4}}^{2}=\sum_{i=1}^{k^{4}}{Ev_{i,1}^{2}}. $$

Obviously, we have

2.21 $$ \sum_{k=1}^{\infty} \bigl(1-\Phi \bigl(1+(1-5\varepsilon) (2\log\log n_{k})^{1/2}\bigr) \bigr)= \infty. $$

Noting that $\{v_{i,1},1\leq i\leq k^{4}\}$ is an LNQD sequence and by (H1), we get

$$\begin{aligned} J_{k,1}&\leq Cn_{k}^{-1}\sum _{2\leq j\leq k^{4}}k^{4}|Ev_{1,1}v_{j,1}| \\ &\leq Cn_{k}^{-1}k^{4}\sum _{1\leq i\leq m_{k}}\sum_{m_{k}+p_{k}\leq j\leq n_{k}}|E \zeta_{i}\zeta_{j}| \\ &\leq C\sum_{p_{k}\leq j\leq n_{k}}|E\zeta_{i} \zeta_{j}|.\end{aligned} $$

By the fact that $n_{k-1}=o(p_{k})$, we see that

2.22 $$ \sum_{k=1}^{\infty}J_{k,1} \leq C\sum_{k=1}^{\infty}\sum _{n_{k-1}\leq j\leq n_{k}}|E\zeta_{i}\zeta_{j}|\leq C. $$

Finally, we estimate $J_{k,2}$. By the Rosenthal type maximal inequality for an LNQD sequence, which can be proved easily as the proof of Theorem 2 from Shao [16], thus we have

$$\begin{aligned} J_{k,2}&\leq Cn_{k}^{-3/2}\sum _{i=1}^{k^{4}}\bigl\{ (m_{k})^{3/2}+m_{k} \bigl(E|\xi_{1}|^{3}I\bigl\{ |\xi_{1}|\leq n_{k}^{1/2}\bigr\} \bigr)+n_{k}^{3/2}P \bigl(|\xi_{1}|>n_{k}^{1/2}\bigr)\bigr\} \\ &\leq C\bigl\{ k^{-2}+n_{k}^{-1/2}E| \xi_{1}|^{3}I\bigl\{ |\xi_{1}|\leq n_{k}^{1/2}\bigr\} +n_{k}P\bigl(|\xi _{1}|>n_{k}^{1/2}\bigr)\bigr\} .\end{aligned} $$

Observe that with $n_{0}=0$

$$\begin{aligned} \sum_{k=1}^{\infty}n_{k}^{-1/2}E| \xi_{1}|^{3}I\bigl\{ |\xi_{1}|\leq n_{k}^{1/2}\bigr\} &=\sum_{k=1}^{\infty}n_{k}^{-1/2} \sum_{j=1}^{k}E|\xi_{1}|^{3}I \bigl\{ n_{j-1}^{1/2}< |\xi_{1}|\leq n_{j}^{1/2}\bigr\} \\ &=\sum_{j=1}^{\infty}\sum _{k=j}^{\infty}n_{k}^{-1/2}E| \xi_{1}|^{3}I\bigl\{ n_{j-1}^{1/2}< | \xi_{1}|\leq n_{j}^{1/2}\bigr\} \\ &\leq C\sum_{j=1}^{\infty}n_{j}^{-1/2}E| \xi_{1}|^{3}I\bigl\{ n_{j-1}^{1/2}< |\xi _{1}|\leq n_{j}^{1/2}\bigr\} \\ &\leq C\sum_{j=1}^{\infty}E| \xi_{1}|^{2}I\bigl\{ n_{j-1}^{1/2}< | \xi_{1}|\leq n_{j}^{1/2}\bigr\} \leq CE| \xi_{1}|^{2}< \infty.\end{aligned} $$

Similarly,

$$ \sum_{k=1}^{\infty}n_{k}P\bigl(| \xi_{1}|>n_{k}^{1/2}\bigr)< \infty. $$

Putting the above inequalities together yields

2.23 $$ \sum_{k=1}^{\infty}J_{k,2}< \infty. $$

This proves (2.19), by combining the above inequalities (2.20)-(2.23). □

## The LIL for linear processes generated by LNQD sequence

In this section, we will discuss the law of iterated logarithm (LIL, for short) for linear processes generated by LNQD sequence with finite variance.

The linear processes are of special importance in time series analysis and they arise in wide variety of concepts (see, *e.g.*, Hannan [20], Chapter 6). Applications to economics, engineering, and physical science are extremely broad and a vast amount of literature is devoted to the study of the theorems for linear processes under various conditions. For the linear processes, Fakhre-Zakeri and Farshidi [21] established CLT under the i.i.d. assumptions and Fakhre-Zakeri and Lee [22] proved a FCLT under the strong mixing conditions. Kim and Baek [23] obtained a central limit theorem for stationary linear processes generated by linearly positively quadrant dependent process. Peligrad and Utev [24] established the central limit theorem for linear processes with dependent innovations including martingales and mixingale. Qiu and Lin [25] discussed the functional central limit theorem for linear processes with strong near-epoch dependent innovations. Dedecker *et al.* [26] provided the invariance principles for linear processes generated by dependent innovations. We will prove the following theorem.

### Theorem 3.1

*Let* $\{\xi_{i},i\in\mathbb{Z}\}$ *be a strictly stationary LNQD sequence with* $E\xi_{i}=0$, $E\xi_{i}^{2}<\infty$ *and* $\sigma^{2}=E\xi_{1}^{2}+2\sum_{i=2}^{\infty}E\xi_{1}\xi_{i}>0$. $\{a_{j},j\in \mathbb{Z}\}$ *be a sequence of real numbers with* $\sum_{j=-\infty}^{\infty}|a_{j}|<\infty$. *Define the linear processes* $X_{t}=\sum_{i=-\infty}^{\infty}a_{i}\xi_{t-i}$. *Then*

3.1 $$ \limsup_{n\to\infty}\frac{|\sum_{t=1}^{n}X_{t}|}{(2\sigma^{2}n\log\log n)^{1/2}}=\Bigg|\sum _{j=-\infty}^{\infty}a_{j}\Bigg| \quad\textit{a.s.} $$

The proof of Theorem 3.1 is based on the following lemmas.

### Lemma 3.2

*Let* $\{\xi_{i},i\in\mathbb{Z}\}$ *be a strictly sequence of random variables*, $\{a_{n},n\geq1\}$ *be a monotone decreasing sequence of nonnegative real numbers*. *Then* $\forall j\in\mathbb{Z}$,

$$ \sup_{n\geq1}\Bigg|a_{n}\sum_{i=1}^{n} \xi_{i-j}\Bigg| \stackrel{\mathrm{d}}{=} \sup_{n\geq1}\Bigg|a_{n} \sum_{i=1}^{n}\xi_{i}\Bigg|. $$

### Proof

Let $Y_{j}=\sup_{n\geq1}|a_{n}\sum_{i=1}^{n}\xi_{i-j}|$, $Y=\sup_{n\geq 1}|a_{n}\sum_{i=1}^{n}\xi_{i}|$. Obviously

$$\begin{aligned}[b] P(Y_{j}\leq x) &=P\Biggl(\bigcap _{k=1}^{\infty}\Biggl({\max_{1\leq t\leq k}\Bigg|a_{t} \sum_{i=1}^{t}\xi_{i-j}\Bigg| \leq x} \Biggr)\Biggr) \\ &= \lim_{k\rightarrow\infty}P\Biggl( \max_{1\leq t\leq k}\Bigg|a_{t} \sum_{i=1}^{t}\xi_{i-j}\Bigg| \leq x \Biggr),\end{aligned} $$

similarly,

$$ P(Y\leq x)=\lim_{k\rightarrow\infty}P\Biggl( \max_{1\leq t\leq k}\Bigg|a_{t} \sum_{i=1}^{t}\xi_{i}\Bigg|\leq x \Biggr). $$

By the strictly stationarity, we know $(\xi_{1-j},\xi_{2-j},\ldots,\xi_{t-j})\stackrel{\mathrm{d}}{=}(\xi _{1},\xi_{2},\ldots,\xi_{t})$, then, for every Borel set $D\in\mathbb{R}^{t}$,

$$ P\bigl\{ (\xi_{1-j},\xi_{2-j},\ldots,\xi_{t-j})\in D\bigr\} =P\bigl\{ (\xi_{1},\xi _{2},\ldots, \xi_{t})\in D\bigr\} . $$

In particular, if we take $D= \{(x_{1},x_{2},\ldots,x_{n}):\max_{1\leq t\leq k}|a_{t}\sum_{i=1}^{t}\xi_{i}|\leq x \}$, then the result of Lemma 3.2 can be obtained by the above statements. □

### Lemma 3.3

*Let* $\{\xi_{i}, i\in \mathbb{Z}\}$ *be a strictly stationary LNQD sequence of random variables with* $E\xi_{1}=0$, $E{\xi_{1}}^{2}<\infty$, ${\sigma}^{2}=E{\xi_{1}}^{2}+2\sum_{i=2}^{\infty} E\xi_{1}\xi_{i}>0$. *Then*

3.2 $$ E\sup_{n}(2n\log\log{n})^{-\frac{1}{2}}\Bigg|\sum _{k=1}^{n}\xi_{k}\Bigg|< \infty. $$

### Proof

Let $b_{n}=(2n\log\log{n})^{\frac {1}{2}}$, ${b_{2^{k}}}/{b_{2^{k+1}}}\rightarrow \frac{\sqrt{2}}{2}$ ($k\rightarrow \infty$), then there exists $C_{1}>0$, such that for all $k\geq 0$, ${b_{2^{k}}}/{b_{2^{k+1}}}\geq C_{1}$. Let *m*, $\sigma_{m}^{2}$, $a_{i}$, $g_{l}(a_{i},\xi_{i})$, $Y_{i,l}$, $S_{i,l}$, $u_{i}$, $U_{i}$ be defined as in the proof of Theorem 2.1. Note that $\sum_{k=1}^{n}\xi_{k}=S_{n,1}+S_{n,2}$. Then

3.3 $$ \begin{aligned}[b] &E\sup_{n}(2n\log\log{n})^{-\frac{1}{2}}\Bigg|\sum _{k=1}^{n}\xi_{k}\Bigg| \\ &\quad\leq \sup_{n}(2n\log\log{n})^{-\frac{1}{2}}|S_{n,1}|+ E\sup_{n}(2n\log\log{n})^{-\frac{1}{2}}|S_{n,2}|.\end{aligned} $$

In order to prove (3.2), it is sufficient to prove

3.4 $$\begin{aligned}& E\sup_{n}(2n\log\log{n})^{-\frac{1}{2}}|S_{n,1}|< \infty, \end{aligned}$$

3.5 $$\begin{aligned}& E\sup_{n}(2n\log\log{n})^{-\frac{1}{2}}|S_{n,2}|< \infty. \end{aligned}$$

Note that

3.6 $$ \begin{aligned}[b] &E\sup_{n}(2n\log \log{n})^{-\frac{1}{2}}|S_{n,2}| \\ &\quad= E\sup_{n}(2n\log\log{n})^{-\frac{1}{2}}\Bigg|\sum _{k=1}^{n} \bigl(g_{2}(a_{k}, \xi_{k})-Eg_{2}(a_{k},\xi_{k})\bigr)\Bigg| \\ &\quad\leq E\sum_{k=1}^{\infty} \frac {|g_{2}(a_{k},\xi_{k})-Eg_{2}(a_{k},\xi_{k})|}{ (2k\log\log{k})^{\frac{1}{2}}} \leq 4\sum_{k=1}^{\infty} \frac{E|\xi_{k}|I{\{|\xi_{k}| >a_{k}\}}}{ (2k\log\log{k})^{\frac{1}{2}}} \\ &\quad\leq CE\xi_{1}^{2}< \infty.\end{aligned} $$

The last inequalities can be induced by the same argument as in (2.11).

Finally, in order to prove (3.2), it remains to check that (3.4) holds by combining the above inequalities. We have

3.7 $$ \begin{aligned}[b] &E\sup_{n}(2n\log \log{n})^{-\frac{1}{2}}|S_{n,1}| \\ &\quad=E\sup_{k\geq0}\max_{2^{k}\leq n< 2^{k+1}} \frac{|S_{n,1}|}{(2n\log\log{n})^{\frac{1}{2}}} \\ &\quad={ \int_{0}}^{\infty}P\biggl\{ \sup_{k\geq0} \max_{2^{k}\leq n< 2^{k+1}} \frac{|S_{n,1}|}{(2n\log\log{n})^{\frac{1}{2}}}>x\biggr\} \,dx \\ &\quad\leq B+{ \int_{B}}^{\infty}P\biggl\{ \sup_{k\geq 0} \max_{2^{k}\leq n< 2^{k+1}} \frac{|S_{n,1}|}{(2n\log\log{n})^{\frac{1}{2}}}>x\biggr\} \,dx,\end{aligned} $$

where *B* will be given later. Noting the choice of $C_{1}$, we have

3.8 $$\begin{aligned} &{ \int_{B}}^{\infty}P\biggl\{ \sup_{k\geq0} \max_{2^{k}\leq n< 2^{k+1}} \frac{|S_{n,1}|}{(2n\log\log{n})^{\frac{1}{2}}}>x\biggr\} \,dx \\ &\quad\leq{ \int_{B}}^{\infty}\sum_{k=0}^{\infty} P\biggl\{ \max_{2^{k}\leq n< 2^{k+1}} \frac{|S_{n,1}|}{(2n\log\log{n})^{\frac{1}{2}}}>x\biggr\} \,dx \\ &\quad\leq\sum_{k=0}^{\infty}{ \int_{B}}^{\infty} P\Bigl\{ \max_{2^{k}\leq n< 2^{k+1}} {|S_{n,1}|}>x\bigl(2\cdot2^{k}\cdot \log\log{2^{k}} \bigr)^{\frac{1}{2}}\Bigr\} \,dx \\ &\quad\leq\sum_{k=0}^{\infty}{ \int_{B}}^{\infty} P\Bigl\{ \max_{1\leq n\leq 2^{k+1}} {|S_{n,1}|}>xC_{1}\bigl(2\cdot2^{k+1}\cdot\log\log {2^{k+1}}\bigr)^{\frac{1}{2}}\Bigr\} \,dx. \end{aligned}$$

It is easy to check that

3.9 $$ \begin{aligned}[b] \max_{n\leq2^{k+1}}|S_{n,1}| &\leq \max_{1\leq i\leq[2^{k+1}/m]}|U_{i}|+ \max_{1\leq i\leq [2^{k+1}/m]}\sum _{j=(i-1)m+1}^{\min(2^{k+1},im)}|Y_{j,1}| \\ &\leq\max_{1\leq i\leq[2^{k+1}/m]}|U_{i}|+2ma_{2^{k+1}} \\ &\leq\max_{1\leq i\leq [2^{k+1}/m]}|U_{i}|+2\varepsilon\sigma \bigl(2^{k+1}\cdot\log\log{2^{k+1}}\bigr)^{\frac{1}{2}}.\end{aligned} $$

By the same argument as of (2.14), there exists $k_{0}$, such that, for every $k\geq k_{0}$,

3.10 $$ \sum_{i=1}^{[2^{k+1}/m]}E{u_{i}}^{2} \leq{\sigma}^{2}\cdot(1+2\varepsilon )\cdot 2^{k+1}. $$

By the definition of LNQD and Lemma 2.3, we know $\{u_{i},i\geq1\} $ are also LNQD random variables with $Eu_{i}=0$ and $|u_{i}|\leq2ma_{im}$ for every *i*. By Lemma 2.4 (with $\alpha =1-\varepsilon$, $a=2ma_{2^{k+1}}$), then by (3.9) and (3.10), observing that $0<\varepsilon<\frac{\sqrt{2}C_{1}}{2\sigma}$, we have

3.11 $$ \begin{aligned}[b] &\sum_{k=0}^{\infty}{ \int_{B}}^{\infty} P\Bigl\{ \max_{1\leq n\leq 2^{k+1}} {|S_{n,1}|}>xC_{1}\bigl(2\cdot2^{k+1}\cdot\log\log {2^{k+1}}\bigr)^{\frac{1}{2}}\Bigr\} \,dx \\ &\quad\leq\sum_{k=0}^{\infty}{ \int_{B}}^{\infty} P\Bigl\{ \max_{1\leq i\leq[2^{k+1}/m]} {|U_{i}|}>(xC_{1}\sqrt{2}-2\varepsilon\sigma) \bigl(2^{k+1}\cdot\log\log {2^{k+1}}\bigr)^{\frac{1}{2}} \Bigr\} \,dx \\ &\quad\leq\sum_{k=0}^{\infty}{ \int_{B}}^{\infty} P\Bigl\{ \max_{1\leq i\leq[2^{k+1}/m]} {|U_{i}|}>(C_{1}\sqrt{2}-2\varepsilon\sigma)x \bigl(2^{k+1}\cdot\log\log {2^{k+1}}\bigr)^{\frac{1}{2}} \Bigr\} \,dx \\ &\quad\leq \frac{2}{\varepsilon}\sum_{k=0}^{\infty}{ \int_{B}}^{\infty} \exp \biggl(-\frac{(C_{1}\sqrt{2}-2\varepsilon\sigma)^{2}(1-\varepsilon )(2^{k+1}\cdot \log\log{2^{k+1}})x^{2}}{4\varepsilon\sigma \cdot2^{k+1}(C_{1}\sqrt{2}-2\varepsilon\sigma)x+ 2\sum_{i=1}^{[2^{k+1}/m]}E{u_{i}}^{2}} \biggr)\,dx \\ &\quad\leq \frac{2}{\varepsilon}\sum_{k=0}^{k_{0}}{ \int_{B}}^{\infty} e^{-A_{k}x}\,dx+ \frac{2}{\varepsilon} \sum_{k=k_{0}+1}^{\infty}{ \int_{B}}^{\infty} e^{-D\cdot(\log\log{2^{k+1}})\cdot x}\,dx \\ &\quad=\frac{2}{\varepsilon}\sum_{k=0}^{k_{0}}{ \int_{B}}^{\infty} e^{-A_{k}x}\,dx+\frac{2}{\varepsilon D} \sum_{k=k_{0}+1}^{\infty} \frac{1}{\log{((k+1)\log{2})}((k+1)\log{2})^{BD}} < \infty,\end{aligned} $$

where

$$\begin{gathered} A_{k}=\frac{(C_{1}\sqrt{2}-2\varepsilon\sigma)^{2}(1-\varepsilon )(2^{k+1}\cdot \log\log{2^{k+1}})}{4\varepsilon\sigma \cdot2^{k+1}(C_{1}\sqrt{2}-2\varepsilon\sigma)+ 2\sum_{i=1}^{[2^{k+1}/m]}E{u_{i}}^{2}}>0, \\ D=\frac{(C_{1}\sqrt{2}-2\varepsilon\sigma)^{2}(1-\varepsilon)}{ 4\varepsilon\sigma (C_{1}\sqrt{2}-2\varepsilon\sigma)+2{\sigma}^{2}\cdot (1+2\varepsilon)}>0,\end{gathered} $$

and choose *B* sufficiently large such that $BD>1$. Thus (3.4) holds by combining the above inequalities together. □

### Proof of Theorem 3.1

By a Beveridge and Nelson decomposition for a linear process, for $m,n,t\in\mathbb{N}$, let

$$\begin{gathered} Y_{m,n}=(2n\log\log{n})^{-\frac{1}{2}}\sum _{t=1}^{n}\sum _{j=-m}^{m} a_{j} \xi_{t-j}, \\ \widetilde{a}_{m}=0, \qquad\widetilde{a}_{j}=\sum _{i=j+1}^{m}a_{i},\quad j=0,1,\ldots,m-1, \\ \widetilde{\widetilde{a}}_{-m}=0,\qquad \widetilde{ \widetilde{a}}_{j} =\sum_{i=-m}^{j-1}a_{i}, \quad j=-m+1,-m+2,\ldots,0, \\ \widetilde{\xi}_{t}=\sum_{j=0}^{m} \widetilde{a}_{j}\xi_{t-j}, \quad\quad \widetilde{\widetilde{ \xi}}_{t}=\sum_{j=-m}^{0} \widetilde{\widetilde{a}}_{j} \xi_{t-j}. \end{gathered} $$

Obviously

3.12 $$\begin{aligned}& Y_{m,n}=\Biggl(\sum_{j=-m}^{m} a_{j}\Biggr) (2n\log\log{n})^{-\frac{1}{2}}\Biggl(\sum _{t=1}^{n}\xi_{t}\Biggr)+ (2n\log \log{n})^{-\frac{1}{2}}(\widetilde{\xi}_{0} -\widetilde{ \xi}_{n}+\widetilde{\widetilde{\xi}}_{n+1}- \widetilde{ \widetilde{\xi}}_{1}), \end{aligned}$$

3.13 $$\begin{aligned}& (2n\log\log{n})^{-\frac{1}{2}}\sum _{t=1}^{n}X_{t}=Y_{m,n}+(2n\log \log {n})^{-\frac{1}{2}} \Biggl(\sum_{t=1}^{n} \sum_{|j|>m}a_{j}\xi_{t-j} \Biggr). \end{aligned}$$

By the strictly stationarity, for every $\varepsilon>0$, we have

3.14 $$ \sum_{n=1}^{\infty}P\bigl\{ | \xi_{n-j}|/(2n\log\log{n})^{\frac{1}{2}}> \varepsilon\bigr\} \leq\sum _{n=1}^{\infty}P\bigl\{ |\xi_{0}|^{2}> 2{\varepsilon}^{2} n\log\log{n}\bigr\} \leq C E|\xi_{0}|^{2}< \infty. $$

Then by the Borel-Cantelli lemma, for any $j\geq0$,

$$ (2n\log\log{n})^{-\frac{1}{2}}\xi_{n-j}\rightarrow0 \quad\text{a.s. } n \rightarrow\infty. $$

Therefore

$$ (2n\log\log{n})^{-\frac{1}{2}} \cdot\widetilde{\xi}_{n}=(2n\log \log{n})^{-\frac{1}{2}}\sum_{j=0}^{m} \widetilde{{a}}_{j} \xi_{n-j} \rightarrow0 \quad\text{a.s. } n \rightarrow\infty. $$

Similarly, we obtain

$$\begin{gathered} (2n\log\log{n})^{-\frac{1}{2}} \cdot\widetilde{\xi}_{0}\rightarrow0 \quad\text{a.s. } n\rightarrow\infty, \\(2n\log\log{n})^{-\frac{1}{2}} \cdot\widetilde{\widetilde{\xi}}_{1} \rightarrow0 \quad\text{a.s. } n\rightarrow\infty, \\(2n\log\log{n})^{-\frac{1}{2}} \cdot\widetilde{\widetilde{\xi}}_{n+1} \rightarrow0 \quad\text{a.s. } n\rightarrow\infty.\end{gathered} $$

By the above statement, we have

3.15 $$ (2n\log\log{n})^{-\frac{1}{2}} (\widetilde{\xi}_{0} - \widetilde{\xi}_{n}+\widetilde{\widetilde{\xi}}_{n+1}- \widetilde{\widetilde{\xi}}_{1})\rightarrow0 \quad\text{a.s. } n \rightarrow\infty. $$

By Theorem 2.1

$$ \limsup_{n\rightarrow\infty}(2n\log\log{n})^{-\frac{1}{2}} \sum _{t=1}^{n}{\xi}_{t}=\sigma \quad \text{a.s.} $$

From the definition of LNQD and Lemma 2.3, it is easy to check that $\{-\xi_{i};i\in\mathbb{Z}\}$ is an LNQD sequence of random variables. Then, by Theorem 2.1,

$$ \limsup_{n\rightarrow\infty}(2n\log\log{n})^{-\frac{1}{2}} \sum _{t=1}^{n}{(-\xi}_{t})=\sigma \quad \text{a.s.} $$

Thus

3.16 $$ \limsup_{n\rightarrow\infty}(2n\log\log{n})^{-\frac{1}{2}} \Bigg| \sum_{t=1}^{n}{\xi}_{t}\Bigg|=\sigma \quad\text{a.s.} $$

Let $S_{n}=\sum_{t=1}^{n}X_{t}$, combining (3.12)-(3.16), then

3.17 $$ \begin{aligned}[b] &\limsup_{n\rightarrow \infty}(2n\log \log{n})^{-\frac{1}{2}} |S_{n}| \\ &\quad=\limsup_{n\rightarrow \infty}\Bigg|Y_{m,n}+\sum _{|j|>m}a_{j} (2n\log\log{n})^{-\frac{1}{2}}\sum _{t=1}^{n}{\xi}_{t-j}\Bigg| \\ &\quad\leq\limsup_{n\rightarrow \infty}\Bigg|\sum _{j=-m}^{m}a_{j}\Bigg| (2n\log \log{n})^{-\frac{1}{2}}\Bigg|\sum_{t=1}^{n}{ \xi}_{t}\Bigg| \\ &\qquad+\limsup_{n\rightarrow\infty} \sum_{|j|>m}|a_{j}|(2n \log\log{n})^{-\frac{1}{2}}\Bigg|\sum_{t=1}^{n}{ \xi}_{t-j}\Bigg| \\ &\quad\leq\Bigg|\sum_{j=-m}^{m}a_{j}\Bigg| \sigma+ \sum_{|j|>m}|a_{j}|\sup _{n}(2n\log\log{n})^{-\frac{1}{2}}\Bigg|\sum _{t=1}^{n}{\xi}_{t-j}\Bigg| \quad\text{a.s.}\end{aligned} $$

Then by the strictly stationarity, Lemma 3.2 and Lemma 3.3, we know

3.18 $$ E\sup_{n}(2n\log\log{n})^{-\frac{1}{2}}\Bigg| \sum _{t=1}^{n}{\xi}_{t-j}\Bigg| =E\sup _{n}(2n\log\log{n})^{-\frac{1}{2}}\Bigg| \sum _{t=1}^{n}{\xi}_{t}\Bigg|< \infty. $$

Then, by (3.18),

3.19 $$ \sup_{n}(2n\log\log{n})^{-\frac{1}{2}}\Bigg| \sum _{t=1}^{n}{\xi}_{t-j}\Bigg|< \infty \quad\text{a.s.} $$

By (3.19), letting $m\to\infty$ in (3.17), we have

3.20 $$ \limsup_{n\rightarrow\infty}(2n\log\log{n})^{-\frac{1}{2}} |S_{n}|\leq4\Bigg|\sum_{j=-\infty}^{\infty}a_{j}\Bigg| \sigma \quad\text{a.s.} $$

On the other hand, by (3.13), (3.15) and (3.16), we obtain

3.21 $$ \begin{aligned}[b] &\limsup_{n\rightarrow \infty}(2n\log \log{n})^{-\frac{1}{2}} |S_{n}| \\ &\quad\geq\limsup_{n\rightarrow\infty}\Bigg|\sum _{j=-m}^{m}a_{j}\Bigg|(2n\log \log{n})^{-\frac{1}{2}} \Bigg|\sum_{t=1}^{n}{ \xi}_{t}\Bigg| \\ &\qquad-\lim_{n\rightarrow \infty} (2n\log\log{n})^{-\frac{1}{2}}| \widetilde{\xi}_{0} -\widetilde{\xi}_{n}+\widetilde{ \widetilde{\xi}}_{n+1}- \widetilde{\widetilde{\xi}}_{1}| \\ &\qquad-\sum_{|j|>m}|a_{j}|\sup _{n} (2n\log\log{n})^{-\frac{1}{2}}\Bigg| \sum _{t=1}^{n}{\xi}_{t-j}\Bigg| \\ &\quad=\Bigg|\sum_{j=-m}^{m}a_{j}\Bigg|\sigma -\sum_{|j|>m}|a_{j}|\sup _{n} (2n\log\log{n})^{-\frac{1}{2}}\Bigg|\sum _{t=1}^{n}{\xi}_{t-j}\Bigg| \quad\text{a.s.}\end{aligned} $$

Then, letting $m\to\infty$,

3.22 $$ \limsup_{n\rightarrow\infty}(2n\log\log{n})^{-\frac{1}{2}} |S_{n}|\geq\Bigg|\sum_{j=-\infty}^{\infty}a_{j}\Bigg| \sigma \quad\text{a.s.} $$

Hence from (3.20) and (3.22) the desired conclusion (3.1) follows. □

## Conclusions

In this paper, using the Kolmogorov type maximal inequality and Stein’s method, the law of the iterated logarithm for LNQD sequence is established with less restriction of moment conditions, this improves the results of Choi [11] from $E|\xi _{1}|^{2+\delta}<\infty$ to $E|\xi_{1}|^{2}<\infty$. We also prove the law of the iterated logarithm for a linear process generated by LNQD sequence with the coefficients satisfying $\sum_{i=-\infty}^{\infty}|a_{i}|<\infty$ by the Beveridge and Nelson decomposition, this extends the law of iterated logarithm for a linear process with the innovations from i.i.d. and NA cases to LNQD random variables.
